# Developing and testing inter‐rater reliability of a data collection tool for patient health records on end‐of‐life care of neurological patients in an acute hospital ward

**DOI:** 10.1002/nop2.1789

**Published:** 2023-05-04

**Authors:** Gudrun Jonsdottir, Erna Haraldsdottir, Valgerdur Sigurdardottir, Asta Thoroddsen, Runar Vilhjalmsson, Gudny Bergthora Tryggvadottir, Helga Jonsdottir

**Affiliations:** ^1^ Faculty of Nursing and Midwifery, School of Health Sciences University of Iceland Reykjavik Iceland; ^2^ Landspitali, The National University Hospital of Iceland Reykjavik Iceland; ^3^ Division of Nursing Queen Margaret University Edinburgh UK; ^4^ The Social Science Research Institute University of Iceland Reykjavík Iceland

**Keywords:** end‐of‐life care, face validity, instrument development, inter‐rater reliability, neurological patients, patient health records

## Abstract

**Aim:**

Develop and test a data collection tool—Neurological End‐Of‐Life Care Assessment Tool (NEOLCAT)—for extracting data from patient health records (PHRs) on end‐of‐life care of neurological patients in an acute hospital ward.

**Design:**

Instrument development and inter‐rater reliability (IRR) assessment.

**Method:**

NEOLCAT was constructed from patient care items obtained from clinical guidelines and literature on end‐of‐life care. Expert clinicians reviewed the items. Using percentage agreement and Fleiss' kappa we calculated IRR on 32 nominal items, out of 76 items.

**Results:**

IRR of NEOLCAT showed 89% (range 83%–95%) overall categorical percentage agreement. The Fleiss' kappa categorical coefficient was 0.84 (range 0.71–0.91). There was fair or moderate agreement on six items, and moderate or almost perfect agreement on 26 items.

**Conclusion:**

The NEOLCAT shows promising psychometric properties for studying clinical components of care of neurological patients at the end‐of‐life on an acute hospital ward but could be further developed in future studies.

## 1 INTRODUCTION

Studies that have investigated end‐of‐life care of neurological patients highlight that it is a complex subject (Alonso et al., 2016; Hussain et al., 2018). To shed light on current practice concerning end‐of‐life care of neurological patients in an acute neurological ward, it was decided to retrospectively retrieve data from patient health records (PHRs). The aim of this paper is to describe the design and testing inter‐rater reliability of a data collection tool to map end‐of‐life care for neurological patients in the last 7 days of their life. The last 7 days were selected since it could be expected that within that time frame indicators of impending death of neurological patients should have manifested and could be noted (Hui et al., 2014, 2015). Designing the data collection tool is part of a larger study of diagnosing dying among neurological patients in an acute hospital ward.

## 2 BACKGROUND

The final stage of palliative care or end‐of‐life care refers to healthcare provided in the last weeks and months of life. End‐of‐life care is given to patients when medical treatment to cure their disease has been brought to an end (Cohen‐Mansfield et al., 2018). The ultimate goal is to relieve suffering, for instance by optimizing pain management and providing psychological and social support to assist patients and their next‐of‐kin with managing physical, emotional, social, and spiritual burden of the imminent death (Huskamp et al., 2012). High quality end‐of‐life care requires communication between the patient, healthcare professionals involved in the care, as well as relatives in order to create a shared understanding about the person's values and treatment preferences (Fernando & Hughes, 2019; Gonella et al., 2020; Sinuff et al., 2015; Taffurelli et al., 2020). This communication ultimately leads to an individualized care plan that is consistent with the patient's values and needs, considering what treatments, including assessments and interventions, will or will not be used to manage the symptoms of the life‐threatening disease (Sinuff et al., 2015). Such individualized care, based on highly tuned clinical judgement, using clinical indicators along with experience and clinical wisdom, is paramount for the right course of action in end‐of‐life care (Kennedy et al., 2014).

To shed light on current practice in relation to end‐of‐life care of neurological patients, a retrospective study is attractive. In this situation the main strength of this non‐intrusive research design is that at this delicate point in the patients' and the relatives' lives they are not bothered by data collection (Kaji et al., 2014). PHRs are widely used in retrospective studies more generally. The records can be in both electronic and paper form (Gregory & Radovinsky, 2012). PHRs contain already documented information, which is intended for patient care, not research purposes. However, there are multiple advantages of using PHRs to obtain research data. The most prominent one is that they can provide a large amount of data of clinical significance at relatively little cost, without taking up patients' time or disturbing patients in any way (Gregory & Radovinsky, 2012; Kaji et al., 2014). PHRs in electronic form (EHR) are more reliable than the paper form as they are stored permanently, so there is less chance of losing data. The shortcomings of retrospective studies of PHRs are that the data is already collected and unchangeable, so the researcher cannot influence what information could be documented; there may be incomplete or missing data; specific patient information may be lacking; and there may be difficulties in interpreting or verifying the information (Feder, 2017; Fortney & Steward, 2015; Jansen et al., 2005). There is also variability in the quality of how healthcare professionals originally documented patient information in the PHRs (Cassidy et al., 2002; Gianinazzi et al., 2015; Gregory & Radovinsky, 2012; Kaji et al., 2014).

With regard to extracting the data, the data abstractors may read, interpret, code, and transcribe what is written in the patients' records differently from what was initially intended, resulting in low sensitivity and specificity of the data collected (Jansen et al., 2005; Kaji et al., 2014). The use of standardized data collection methods or guidelines to ensure consistent collection of data enhances the quality of the data and minimizes bias (Gregory & Radovinsky, 2012; Jansen et al., 2005; Kaji et al., 2014). Reporting on how inter‐rater reliability (IRR) in data collection is achieved shows transparency and may avoid inconsistencies in extraction of the data both within and between the data abstractors (Alonso et al., 2016; Cox et al., 2011; Fortney & Steward, 2015; Kaji et al., 2014). Reports on IRR with percentage agreements and kappa calculations from studies where data has been collected from PHRs have shown the data collection tools to be reliable, with high sensitivity and specificity (Gianinazzi et al., 2015; Ntlholang et al., 2016; Yawn & Wollan, 2005).

Several studies of patients with various neurological diseases have been conducted to improve care and provide evidence for the need of person‐centered end‐of‐life care, with some studies using retrospective PHRs (Alonso et al., 2016; Cheng et al., 2017; Hussain et al., 2018; Liu et al., 2017; Munroe et al., 2007; Ntlholang et al., 2016; Quadri et al., 2018; Wang et al., 2018; Williams et al., 2019). These studies provide limited information on the data collection tools that were used, their psychometric properties, and the training of data abstractors.

## 3 DESIGNING A DATA COLLECTION TOOL

To retrospectively study the practice of end‐of‐life care of neurological patients in an acute hospital ward with the use of PHRs, a data collection tool—Neurological End‐Of‐Life Care Assessment Tool (NEOLCAT)—was developed. The aim of developing the NEOLCAT is to capture how and if signs that may indicate imminent death of neurological patients are identified and documented. The NEOLCAT has the potential to aid future research of care of neurological patients at end‐of‐life. Further, the NEOLCAT has the potential to be used in clinical care to support identification of neurological patients who are facing imminent death and thus supporting decision making in relation to shifting the focus of care to end‐of‐life care, thus improving care for this patients' group.

The NEOLCAT (see File S1) contains key components of end‐of‐life care of neurological patients on an acute hospital ward as highlighted in the literature, including demographics/background, dates of major decisions about treatment, clinical signs and symptoms, laboratory and other tests undertaken, medically invasive and other treatments, communication with relatives, and healthcare professionals' contribution to end‐of‐life care.

The development and testing of the NEOLCAT were divided into three phases: (1) *review of the literature*, (2) *expert advice*, and (3) *ensuring and reporting IRR* (Gregory & Radovinsky, 2012; Jansen et al., 2005; Kaji et al., 2014).

### 3.1 Review of literature

This *first phase* included identifying and studying relevant literature and guidelines in the relation to the purpose of the NEOLCAT. A medical librarian assisted with a literature search for the keywords: diagnosing dying, end‐of‐life care, palliative care, terminal care, neurological disease, stroke, ALS, MND, and Parkinson's, which yielded 20 relevant articles and guidelines that informed the NEOLCAT (Alonso et al., 2016; Bruera et al., 2014; Cheng et al., 2017; Cohen‐Mansfield et al., 2018; Eriksson et al., 2016; Highet et al., 2014; Hui et al., 2014, 2015; Huskamp et al., 2012; Kennedy et al., 2014; Landspitali, 2017; Liu et al., 2017; Mazzocato et al., 2010; McCusker et al., 2013; Munroe et al., 2007; National Clinical Guideline Centre (NICE), 2015; Ntlholang et al., 2016; Quadri et al., 2018; Registered Nurses' Association of Ontario (RNAO), 2011; Sinuff et al., 2015). Systematically scrutinizing the literature generated a list of possible items, which was listed in an Excel file. Of notice is the emphasis that is placed on communication with relatives, and on spirituality. Although these are important aspects of end‐of‐life care for neurological patients, the format for documenting them in EHR is yet to be developed which made it unlikely that they would be identified and extracted (Forde‐Johnston et al., 2022; Sjöberg et al., 2021). The spectrum of these items is therefore limited in the NEOLCAT. Table 1 shows literature and guidelines that formed the foundation of the NEOLCAT.

**TABLE 1:** Key literature and clinical guidelines for end‐of‐life care foundational to the NEOLCAT.

	Major treatment decisions	Clinical signs and symptoms	Laboratory and other tests	Medically invasive and other treatments	Communications with relatives	Healthcare professionals' contribution to decision‐making
Alonso et al. (2016)	√	√	√	√		√
Bruera et al. (2014)		√	√			
Cheng et al. (2017)	√	√	√	√		√
Cohen‐Mansfield et al. (2018)		√				
Eriksson et al. (2016)	√	√	√	√	√	√
Highet et al. (2014)	√	√	√	√	√	√
Huskamp et al. (2012)	√	√	√	√	√	√
Hui et al. (2014), Hui et al. (2015)		√				
ISCI/McCusker et al. (2013)	√	√	√	√	√	√
Kennedy et al. (2014)	√	√	√	√	√	√
Landspitali (2009/, 2017	√	√	√	√	√	√
Liu et al. (2017)	√	√				√
Mazzocato et al. (2010)	√	√	√	√		√
Munroe et al. (2007)	√					√
NICE (2015)	√	√	√	√	√	√
Ntlholang et al. (2016)	√	√		√		√
RNAO (2011)	√	√	√	√	√	√
Sinuff et al. (2015)	√	√			√	√
Quadri et al. (2018)	√	√	√	√	√	√

ICSI, Institute for Clinical Systems Improvement; Landspitali, Palliative Care Guidelines; NICE, National Institute for Health and Care Excellence; RNAO, Registered Nurses' Association of Ontario.

### 3.2 Expert advice

The *second phase* consisted of an iterative process of developing the item list further to its completion. This included conversations with professional experts in palliative care, neurology, and health informatics to gain consensus on items that would finally make up the NEOLCAT. With several formal and informal meetings with the experts, the item list was refined based on their comments and suggestions and finally approved. The NEOLCAT has both objective and subjective items. The wording of each item was carefully studied. A decision was made to have the NEOLCAT in English, with translations in Icelandic in the coding manual. The first draft consisted of 84 items and the final version has 76 items falling into the following seven categories: Demographics/Background, major decisions in treatments, clinical signs and symptoms, laboratory and other tests, medically invasive and other treatments, communication with relatives, and health professionals contribution. Finally, the NEOLCAT was inserted in Research Electronic Data Capture (RedCap) (Harris et al., 2009).

### 3.3 Ensuring and reporting the inter‐rater reliability of the NEOLCAT

**FIGURE 1:**
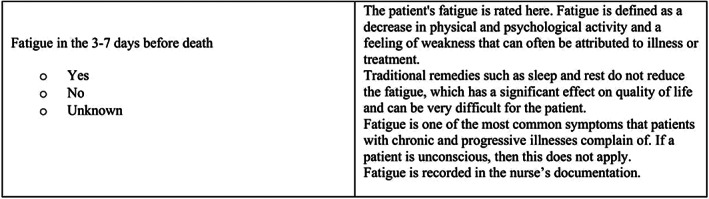
Definition of fatigue. An example from the coding manual.

The *third phase* entailed ensuring a consistent data collection procedure and testing the IRR of the NEOLCAT. A coding manual of definitions of items in the NEOLCAT and how to locate each item in the PHRs was developed. The coding manual was made as accurate as possible with guiding images presented for how and where the data extractors could find the exact information. An example of a definition in the coding manual is given in Figure 1.

The data abstractor team consisted of two students in medicine and psychology and one registered nurse. They underwent individualized training as data abstractors, which consisted of a detailed presentation of the study and instruction in using the coding manual and in extracting the data. Following this, the abstractors' team met formally twice with the primary researcher (GJ) for co‐ordination. Face‐to‐face contacts between the researcher and each of the abstractors continued with numerous meetings during the data collection period. During this training period inter‐rater reliability was calculated three times and the mismatch that was found was explored and corrected. This allowed for discussion around ways of handling conflicting data and clearing up misunderstandings. This was key to facilitating and gaining a shared understanding of each of the items in the NEOLCAT. Examples of issues that needed to be resolved were misunderstandings about the dates of radiological examinations. The study is limited to the last 7 days of life, but the data abstractors, on some occasions, wrongly used information that was documented within a longer range than 7 days. This was corrected after meetings with the researcher. The medium time for extracting data for one patient's EHR was about 60 min.

#### 3.3.1 Ensuring face validity

Face validity was ensured by thoroughly reviewing the literature and having several rounds of communication with the clinical experts, all with over 20 years of experience. When designing an instrument there should be an exhaustive literature review, rich first‐hand knowledge of end‐of‐life care, consultation with experts, and in‐depth conversation with members of the target population, in our case, the healthcare professionals (Polit & Beck, 2017). All available EHRs of patients (*N* = 170) who had died in the acute neurological unit over the five previous years were included in the study.

#### 3.3.2 Testing inter‐rater reliability

Inter‐rater reliability is defined as the degree of agreement or consensus of raters, in this case, the consistency of extracting data with NEOLCAT by data abstractors (Landis & Koch, 1977; LeBreton & Senter, 2008; McHugh, 2012; Polit & Beck, 2017). It was established by regular communication and meetings between researchers and data abstractors to discuss and handle conflicting data from the patient records and evaluated by calculating percentage agreement and the Fleiss' kappa coefficient. The Fleiss' kappa coefficient was categorized into fair (0.21–0.40), moderate (0.41–0.60), substantial (0.61–0.80), and almost perfect agreement (0.81–1.00); (Landis & Koch, 1977). There should be a consistency between high percentage agreement and high Fleiss' kappa coefficient. Therefore, it is important to present both calculations (Feinstein & Cicchetti, 1990; To et al., 2008).

Percentage agreement was calculated without delay in the study period as soon as three abstractors had finished abstracting the same PHRs. All three data abstractors extracted data from eight randomly selected PHRs, subsequently calculating the percentage agreement and the Fleiss' kappa, considering agreement occurring by chance (Cassidy et al., 2002; Feinstein & Cicchetti, 1990; Landis & Koch, 1977; Polit & Beck, 2017; To et al., 2008; Zaiontz, 2019). A percentage agreement that was under 80% was given special consideration and action was taken to rectify data extraction on that item. Disagreement decreased substantially after issues about data extraction of the first three PHRs had been resolved. The data that was gained in the last round of rectifying the data extraction is used in this paper.

With multiple data abstractors it is possible that IRR can be compromised (Gianinazzi et al., 2015). Gregory and Radovinsky (2012) highlighted that to obtain high IRR it is necessary to conduct continuous monitoring and periodic reviews, and to make detailed reports on limitations that were encountered through the data abstraction processes, and to explain how these limitations were addressed (Gregory & Radovinsky, 2012). The items chosen for IRR calculation were nominal variables having yes/no/unknown coding possibilities. Thirty‐two items out of 76 were chosen by importance. Items with the possibility of multiple answers e.g., medical diagnoses, signs of dying, and text answers were not included. In calculating percentage agreement and Fleiss' kappa coefficients, two categories were grouped together, Demographic and Major decisions on treatments, since both contained few items.

## 4 RESULTS

We report on average percentage agreement and average Fleiss' kappa coefficient for the 32 items of the NEOLCAT belonging to six categories having one to 16 items each, see Table 2 (see supplement 1 for the total of categories and items). This is followed by calculations of congruence between the NEOLCAT items into fair, moderate, substantial, and almost perfect agreement.

**TABLE 2:** Percentage agreements and Fleiss' kappa coefficients.

Items	Percentage agreement	Fleiss' kappa
**Demographics/background**		
Number of admittances to the hospital	92	0.89
**Major decisions in treatment**		
Use of “Care pathway for the dying”	92	0.74
**Clinical signs and symptoms**		
Assessment of consciousness 48 h before death	75	0.50
Assessment of consciousness 3–7 days before death	83	0.75
Assessment of alertness/responsiveness 48 h before death	83	0.24
Assessment of alertness /responsiveness in 3–7 days before death	100	1.00
Signs of progression of disease in the 48 h before death	92	0.80
Signs of progression of disease in the 3–7 h before death	92	0.70
Assessment of mobility 48 h	100	1.00
Assessment of mobility 3–7 days	75	0.58
Fatigue in the 48 h before death	75	0.43
Fatigue in the 3–7 days before death	100	1.00
Nausea in the 48 h before death	83	0.82
Nausea in the 3–7 days before death	83	0.72
Dyspnoea in the 48 h before death	71	0.59
Dyspnoea in the 3–7 days before death	71	0.73
Fluid balance in the 48 h before death	100	1.00
Fluid balance in the 3–7 days before death	100	1.00
**Laboratory and other tests undertaken**		
Blood tests in the 48 h before death	83	0.60
Urine tests taken in the 48 h before death	83	0.75
Urine culture taken in the last 7 days	92	0.88
**Medically invasive and other treatments**		
Vital signs measured in the 48 h before death	100	1.00
Vital signs measured in the 3–7 days before death	92	0.76
Intravenous catheter in place at the time of death	88	0.80
Urinary catheter in place at the time of death	83	0.73
Antibiotics given in the last 48 h	100	1.00
Pain medication given in the last seven days	100	1.00
Tube feeding given in the last seven days	100	1.00
**Communication with relatives**		
Relatives mentioned in PHRs	100	1.00
Relatives present at the time of death of the patient	92	0.83
Health professionals meeting with relatives prior to death (family meeting, formal or informal)	92	0.83
**Health professionals' contribution to decision‐making of end‐of‐ life care**		
Evidence of health care professionals' assessment of patients' needs for end‐of‐life care	83	0.66
Overall percentage and Fleiss' kappa	89%	0.84

### 4.1 Percentage agreement

Percentage agreement on the 32 items of NEOLCAT varied from 71% to 100% with 89% overall percentage agreement, see Table 3. The percentage agreement of the six categories ranged between 83% and 95%. The category Health professionals' contribution (83%) was the lowest and Medically invasive and other treatments the highest (95%). The percentage agreement range was biggest in the category Clinical signs and symptoms (71%–100%).

**TABLE 3:** Inter‐rater reliability.

	Number of items	Percent agreement		Kappa	
		Category (%)	Range (%)	Category K (95% CI)	K range across items
Demographics and major treatment decisions	2	88	83–92	0.85 (0.73–0.97)	0.76–0.86
Clinical signs and symptoms	16	86	71–100	0.83 (0.76–0.89)	0.24–1.00
Laboratory and other tests undertaken	3	86	83–92	0.71 (0.48–0.95)	0.40–0.83
Medically invasive & other treatments	7	95	83–100	0.91 (0.90–0.93)	0.43–1.00
Communication with relatives	3	94	92–100	nc	
Health professionals' contribution	1	83	83	nc	
Overall	32	89		0.84 (0.80–0.89)	

nc, Not calculated because of small distribution in the sample.

### 4.2 Fleiss' kappa coefficient

The Fleiss' kappa coefficient of the 32 items varied between 0.24 and 1.0 with overall coefficient 0.84, see Table 3. The coefficients of the six categories ranged between 0.71 and 0.91. It was highest in the category Medically invasive and other treatments (range 0.43–1.0) and lowest in the category Clinical signs and symptoms (range 0.24–1.0) which had also the biggest range.

### 4.3 Congruence between percentage agreement and Fleiss' kappa coefficient

Congruence between percentage agreement and Fleiss' kappa coefficient is presented on all the 32 items.

#### 4.3.1 Fair congruence

There was a fair agreement for two items: *Alertness*/*responsiveness* and *fatigue in the last 48 h before death*. In *alertness*/*responsiveness at 48 h before death*, the percentage agreement was 83% and Fleiss' kappa coefficient 0.24. For *fatigue in the last 48 h*, the percentage agreement was 75% and kappa coefficient 0.43. The low kappa coefficient and high percentage agreement contradict each other.

#### 4.3.2 Moderate congruence

There was moderate percentage agreement for four items: *Consciousness*, *dyspnea*, *blood tests at 48 h*, and *mobility in the 3–7 days before death*. The percentage agreement was ranged from 71% to 83%. The kappa coefficient was reasonably consequent and ranged from 0.50 to 0.60.

#### 4.3.3 Substantial congruence

There was substantial agreement for 11 items: *Progression of disease* both at 3–7 days and 48 h before death, *consciousness* in 3–7 days, *dyspnea and nausea* in 3–7 days, *vital signs* in 3–7 days, *urine tests*, *intravenous catheter*, *urinary catheter*, *care pathway for the dying* and *assessment of need for end‐of‐life care by health professional*. The percentage agreement ranged between 71% and 100%. The kappa coefficient was consequent and ranged from 0.66 to 0.80.

#### 4.3.4 Almost perfect congruence

*There was almost perfect agreement for 15 items: Number of admissions*, *alertness/responsiveness at 3–7 days before death*, *vital signs* and *mobility at 48 h*, *nausea at 48 h*, *fatigue at 3–7 days*, *fluid assessment at 48 h and 3–7 days*, *urine culture*, *antibiotics*, *pain medication*, *tube feeding*, *relatives mentioned in PHRs*, *relative's presence at time of death* and *health professionals meeting with relatives prior to death*. The percentage agreement ranged between 83% and 100%. The kappa coefficient was consequent and ranged from 0.82 to 1.00.

## 5 DISCUSSION

Findings of this study show that the NEOLCAT data collection tool which was developed to retrospectively collect data from PHRs for research on end‐of‐life care for neurological patients in an acute hospital ward shows promising psychometric properties. We established face validity in close collaboration with clinical experts and followed guidelines in the literature to enhance accuracy of data extraction. The consistency in the work of the data abstractors was established by calculating inter‐rater reliability (IRR). The overall categorical percentage agreement of the NEOLCAT was 89% (range 83%–95%) and overall Fleiss's kappa categorical coefficient was 0.84 (range 0.71 to 0.91). This shows that NEOLCAT has high inter‐rater reliability and suggests that it is of acceptable quality to be used in our larger study of end‐of‐life care of neurological patients in an acute hospital ward.

There was fair congruence between two items and moderate congruence between four. Incongruence could be expected on a few items and can be explained by lack of rigour in interpreting clinical information in free text and difficulties in locating items in the PHRs. The category with the lowest percentage agreement had only one item and the data were to be extracted from free text. Items of the category Laboratory and other tests undertaken showed acceptable but lower percentage agreement and Fleiss' kappa coefficient than could be expected since required interpretation is minimal. This data was, however, to be found in free text rather than having a fixed location in PHR, which may explain the low values that were found. Several of the items in the category, Clinical signs and symptoms (12 items), required interpretation of free text, yet did have acceptable values. It is concluded that overall, the IRR was acceptable. Low percentage agreement and Fleiss' kappa coefficient would to a great extent be explained by lack of rigorousness of the guidelines in the coding manual as to how to interpret clinical information and where to locate data. The coding manual, therefore, needs revision on those items.

In this study, it was not possible to extract data from PHRs of all the patients simultaneously from the data warehouse. For that to be possible, the PHR needs to have a more rigorous structure and built into the coding system. Therefore, we manually extracted data for one patient at a time, which is both time‐consuming and increases likelihood of mistakes. An important implication of this study is, therefore, the need for improvements in how the PHR is structured and coded.

The general assumption is that end‐of‐life care involves teamwork (Fernando & Hughes, 2019; Kennedy et al., 2014). Teamwork was not reflected in the PHR. Nurses, physicians, and other healthcare professionals wrote in different sections of the PHR about the same issues of the care that was provided. It would be useful to have a structured and coded PHR jointly for all healthcare professionals when documenting end‐of‐life care. That would not only improve the quality of documented care and identify areas for improvement in clinical practice but would also ease data collection in future research.

### 5.1 Strength and limitations

It is a considerable strength of the study to have substantiative literature for item selection and expert advice from different healthcare professionals to contribute to the design of the NEOLCAT. There were however only a couple of instruments on which to ground the NEOLCAT. Another strength is the rigorousness of the data extraction process, the training of the data abstractors and the use of a coding manual.

There are inbuilt limitations to data collection tools that are aimed for researching PHR, most importantly that the research data is unchangeable and that they are intended for patient care, not research purposes. Lack of structure and coding possibilities of the PHR is a limitation as well. Items of communication with patient and family, and on spirituality, in the NEOLCAT are incomplete. There should be a strong focus on these issues in future development of the NEOLCAT.

## 6 CONCLUSIONS

The NEOLCAT data collection tool which was developed to retrospectively collect data from PHR for research on end‐of‐life care for neurological patients in an acute hospital ward shows promising psychometric properties and has the potential to be used in clinical research. It contains items of patient demographics, dates of major treatment decisions, clinical signs and symptoms, laboratory and other tests, medically invasive and other treatments, communication with relatives, and healthcare professionals' involvement in end‐of‐life care. Items of communication with patient and family and on spirituality are incomplete in the NEOLCAT. Those items should be advanced in future development of the NEOLCAT.

## AUTHOR CONTRIBUTIONS

GJ, EH, and HJ were responsible for the study conception and design. RV and GBT supervised the statistical analysis. GJ, EH, VS, AT, RV, GBT, and HJ worked collectively on interpretation of data. GJ was responsible for drafting the manuscript and all the co‐authors provided ideas for improvement.

## CONFLICT OF INTEREST STATEMENT

The authors declare no conflicts of interest.

## FUNDING INFORMATION

This study was supported by the grants received by GJ from the following institutions: The Icelandic Nurses´ Association; The University of Iceland and Landspitali, The National University Hospital of Iceland. The funding sources were not involved in the data analysis and interpretation of results.

## ETHICAL APPROVAL

The Ethics Committee at Landspitali National University Hospital approved the study with the number #26/2017. No identifiable patient information was used, thus, informed consent was not needed.

## Supporting information


File S1

## Data Availability

The data that support the findings of this study are available from the corresponding author upon reasonable request.
