# Supplementary material for: Developing and testing inter‐rater reliability of a data collection tool for patient health records on end‐of‐life care of neurological patients in an acute hospital ward
Source: Nurs Open. 2023 May 4;10(8):5500–8. doi: 10.1002/nop2.1789 (PMC10333872; doi:10.1002/nop2.1789)
Supplement: Supplementary file 1 — File S1 [file NOP2-10-5500-s001.pdf]

Supplementary file 1: Neurological End-of-life Care Assessment Tool (NEOLCAT) data collection tool on end-of-life care of neurological patients in an acute hospital ward.

1. Demographics/Background

ID number | **Date and time of death** | **Age at death**

Gender

☐ 1. Male ☐ 2. Female ☐ 3. Other

Marital status

☐ 1.Married ☐ 2. Single ☐ 3. Divorced ☐ 4. Widowed ☐ 5. Unknown

Neurological medical diagnosis

☐ 1. MND/ALS
☐ 2. Parkinson and extrapyramidaldiseases
☐ 3. Tumour
☐ 4. Stroke - ischaemic I 61- I 63
☐ 5. Stroke - haemorrhage I 60
☐ 6. Other neurological diagnoses

Cause of death *(from death certificate)*

Housing

☐ 1. Living at home ☐ 2. Service apartment ☐ 3. Nursing home ☐ 4. Other

Living arrangements at home:

☐ 1. Alone ☐ 2. With spouse ☐ 3. With relatives ☐ 4. Other

Days in hospital in the previous year

Number of admittances to the hospital in the previous year

Number of admittances to emergency care in previous year

Length of stay in hospital before death

Type of room in ward at time of death ☐ 1. Single room ☐ 2. Shared with others ☐ 3. No information

2. Major decisions in treatment

Treatment directives at end of life

☐ 1. Full treatment
☐ 2. Full treatment but not cardiopulmonary resuscitation, or limited treatment
☐ 4. End of life care
☐ 5. No documentation

Date of documentation of end-of-life care

Use of "*Care pathway for the dying*"

☐ 1. Yes ☐ 2. No ☐ 3. Unknown

Date and time of "*Care pathway for the dying*"

3. Clinical signs and symptoms

3-7 days before death

Consciousness assessment

☐ 1. Full consciousness
☐ 2. Limited consciousness
☐ 3. Unconscious
☐ 4. Unknown

Communication with patient

☐ 1. Responsive
☐ 2. Not responsive
☐ 3. Unknown

Functional assessment

☐ 1. Moving without assistance
☐ 2. Needs assistance with movement
☐ 3. Bedbound
☐ 4. Unknown

Signs of progression of disease

☐ 1. No, patient stable
☐ 2. Yes, patient shows progression
☐ 3. Unknown

Clinical signs of death approaching

☐ 1. Cheyne-Stokes breathing (irregular breathing)
☐ 2. Pulselessness of the radial artery
☐ 3. Decreased urine output
☐ 4. Respiration with mandibular movement
☐ 5. Death rattle
☐ 6. Decreased response to verbal stimuli
☐ 7. Decreased response to visual stimuli
☐ 8. Hyperextension of neck
☐ 9. Drooping of nasolabial fold
☐ 10. Non-reactive pupil
☐ 11. Grunting of vocal cords
☐ 12. Inability to close eyelids
☐ 13. No signs registered

Is the patient comfortable?

☐ 1. Clear indications of systematic assessment of comfort
☐ 2. Clear indications of comfort goals achieved
☐ 3. Unknown

Highest Pain Score

☐ 0	☐ 1	☐ 2
☐ 3	☐ 4	☐ 5
☐ 6	☐ 7	☐ 8
☐ 9	☐ 10	

☐ 11. No documentation/ unknown
☐ 12. Not applicable *(pt. unconscious or unresponsive)*

0-2 days before death

Consciousness assessment

☐ 1. Full consciousness
☐ 2. Limited consciousness
☐ 3. Unconscious
☐ 4. Unknown

Communication with patient

☐ 1. Responsive
☐ 2. Not responsive
☐ 3. Unknown

Functional assessment

☐ 1. Moving without assistance
☐ 2. Needs assistance with movement
☐ 3. Bedbound
☐ 4. Unknown

Signs of progression of disease

☐ 1. No, patient stable
☐ 2. Yes, patient shows progression
☐ 3. Unknown

Clinical signs of death approaching

☐ 1. Cheyne-Stokes breathing (irregular breathing)
☐ 2. Pulselessness of the radial artery
☐ 3. Decreased urine output
☐ 4. Respiration with mandibular movement
☐ 5. Death rattle
☐ 6. Decreased response to verbal stimuli
☐ 7. Decreased response to visual stimuli
☐ 8. Hyperextension of neck
☐ 9. Drooping of nasolabial fold
☐ 10. Non-reactive pupil
☐ 11. Grunting of vocal cords
☐ 12. Inability to close eyelids
☐ 13. No signs registered

Is the patient comfortable?

☐ 1. Clear indications of systematic assessment of comfort
☐ 2. Clear indications of comfort goals achieved
☐ 3. Unknown

Highest Pain Score

☐ 0	☐ 1	☐ 2
☐ 3	☐ 4	☐ 5
☐ 6	☐ 7	☐ 8
☐ 9	☐ 10	

☐ 11. No documentation/ unknown
☐ 12. Not applicable *(pt. unconscious or unresponsive)*

**Pain management**<br><br>☐ 1. Assessment of pain<br>☐ 2. Pain medication given<br>☐ 3. Assessment of pain medication<br>☐ 4. Other treatments of pain<br>☐ 5. Unknown	**Pain management**<br><br>☐ 1. Assessment of pain<br>☐ 2. Pain medication given<br>☐ 3. Assessment of pain medication<br>☐ 4. Other treatments of pain<br>☐ 5. Unknown
**Fatigue**<br><br>☐ 1. Yes ☐ 2. No ☐ 3. Unknown	**Fatigue**<br><br>☐ 1. Yes ☐ 2. No ☐ 3. Unknown
**Nausea**<br><br>☐ 1. Yes ☐ 2. No ☐ 3. Unknown	**Nausea**<br><br>☐ 1. Yes ☐ 2. No ☐ 3. Unknown
**Dyspnoea**<br><br>☐ 1. Yes ☐ 2. No ☐ 3. Unknown	**Dyspnoea**<br><br>☐ 1. Yes ☐ 2. No ☐ 3. Unknown
**Agitation**<br><br>☐ 1. Yes, agitated<br>☐ 2. Intermittent agitation<br>☐ 2. Not agitated<br>☐ 4. Unknown	**Agitation**<br><br>☐ 1. Yes, agitated<br>☐ 2. Intermittent agitation<br>☐ 2. Not agitated<br>☐ 4. Unknown
**Nutrition management**<br><br>☐ 1. Food taken orally<br>☐ 2. Enteral nutrition<br>☐ 3. Parenteral nutrition<br>☐ 4. No food intake<br>☐ 5. Unknown	**Nutrition management**<br><br>☐ 1. Food taken orally<br>☐ 2. Enteral nutrition<br>☐ 3. Parenteral nutrition<br>☐ 4. No food intake<br>☐ 5. Unknown
**Fluid intake**<br><br>☐ 1. Orally<br>☐ 2. Intravenous<br>☐ 3. Fluid via feeding tube<br>☐ 4. No intake<br>☐ 5. Unknown	**Fluid intake**<br><br>☐ 1. Fluid intake orally<br>☐ 2. Intravenous fluids<br>☐ 3. Fluid via feeding tube<br>☐ 4. No fluid intake<br>☐ 5. Unknown
**Vital signs**<br><br>☐ 1. Vital signs measured<br>☐ 2. Vital signs discontinued<br>☐ 3. Unknown	**Vital signs**<br><br>☐ 1. Vital signs measured<br>☐ 2. Vital signs discontinued<br>☐ 3. Unknown

**Body weight**	☐ 1. Stable body weight (+/-1-5 kg)<br>☐ 3. Cachexia	☐ 2. Progressive weight loss (loss of 5-10kg or more)<br>☐ 4. Unknown
**Fall risk assessment (MORSE scale)**	☐ 1. 1-24 no risk<br>☐ 3. 46+ high risk	☐ 2. 25-45 low to moderate risk<br>☐ 4. Unknown
**Discussion with health professionals of end-of-life care – Documentation of *wishes of patient***	☐ 1. Discussion with patient<br>☐ 3. Unknown	☐ 2. Discussion with relatives

**Psychological counselling**<br><br>☐ 1. Offered by staff<br>☐ 2. No counselling<br>☐ 3. Received<br>☐ 4. Unknown	**Social worker's service**<br><br>☐ 1. Offered by staff<br>☐ 2. No counselling<br>☐ 3. Received<br>☐ 4. Unknown
**Chaplain service**	☐ 1. Chaplain services offered by staff<br>☐ 2. No service<br>☐ 3. Service received<br>☐ 4. Unknown
**Documentation of "*Care pathway of the dying*"**	☐ 1. Well documented, goals obtained<br>☐ 2. Well documented, many deviances, goals not or partly obtained<br>☐ 3. Partly documented<br>☐ 4. No documentation<br>☐ 5. Unknown
**Advise sought from palliative care team**	☐ 1. Not documented /unknown ☐ 2. Visit from palliative care team
**Consideration of eligibility for transfer to a palliative care unit?**<br>*(application or referred)*	☐ 1. Yes ☐ 2. No<br>☐ 3. Information inconclusive or not available

4. Laboratory and other tests in the last 7 days before death

Imaging	Reason documented for imaging	Date of last imaging
☐ 1. X-ray<br>☐ 2. CT<br>☐ 3. MRI<br>☐ 4. Ultrasound<br>☐ 5. Other imaging<br>☐ 6. No imaging	☐ 1. Clear reason (routine)<br>☐ 2. Some or partly given reason<br>☐ 3. No reason	
**Blood tests**	**Reason documented for blood tests**	**Date of last blood test**
☐ 1. Yes<br>☐ 2. No	☐ 1. Clear reason (routine)<br>☐ 2. Some or partly given reason<br>☐ 3. No reason	
**Blood culture**	**Reason documented for blood culture**	**Date of last blood culture**
☐ 1. Yes<br>☐ 2. No	☐ 1. Clear reason<br>☐ 2. Some or partly given reason<br>☐ 3. No reason	
**Urine tests**	**Reason documented for urine tests**	**Date of last urine test**
☐ 1. Yes<br>☐ 2. No	☐ 1. Clear reason (routine)<br>☐ 2. Some or partly given reason<br>☐ 3. No reason	
**Urine culture**	**Reason documented for urine culture**	**Date of last urine culture**
☐ 1. Yes<br>☐ 2. No	☐ 1. Clear reason<br>☐ 2. Some or partly given reason<br>☐ 3. No reason	
**Sputum culture**	**Reason documented for sputum culture**	**Date of last sputum culture**
☐ 1. Yes<br>☐ 2. No	☐ 1. Clear reason<br>☐ 2. Some or partly given reason<br>☐ 3. No reason	
**Faeces test**	**Reason documented for testing faeces**	**Date of last faeces test**
☐ 1. Yes<br>☐ 2. No	☐ 1. Clear reason<br>☐ 2. Some or partly given reason<br>☐ 3. No reason	
**Blood sugar tests**	**Reason documented for blood sugar testing**	**Date of last blood sugar test**
☐ 1. Yes<br>☐ 2. No	☐ 1. Clear reason (routine)<br>☐ 2. Some or partly given reason<br>☐ 3. No reason	

Spirometry test ☐ 1. Yes ☐ 2. No ☐ 3. No information available

5. Medically invasive and other treatments in the last day/hours before death

Venflon or CVK in place at death

☐ 1. Yes
☐ 2. No
☐ 3. Not known

If yes, is there a reason documented for Venflon or CVK?

☐ 1. Clear reason
☐ 2. Some or partly given reason
☐ 3. No reason

Feeding tube present at death

☐ 1. Yes
☐ 2. No
☐ 3. Not known

If yes, is there a reason documented for the feeding tube?

☐ 1. Clear reason
☐ 2. Some or partly given reason
☐ 3. No reason given

Urinary catheter present at death

☐ 1. Yes
☐ 2. No
☐ 3. Not known

If yes, is there a reason documented for the urinary catheter

☐ 1. Clear reason
☐ 2. Some or partly given reason
☐ 3. No reason given

Subcutis - butterfly needle present at death

☐ 1. Yes
☐ 2. No
☐ 3. Not known

If yes, is there a reason documented for the butterfly needle

☐ 1. Clear reason
☐ 2. Some or partly given reason
☐ 3. No reason given

Heart rate monitoring at time of death

☐ 1. Yes, heart rate monitor
☐ 2. No monitoring
☐ 3. Not known

If yes, is there a reason documented for the heart rate monitoring?

☐ 1. Yes, a clear reason
☐ 2. Somewhat or partly given reason
☐ 3. No reason given

Antibiotics

☐ 1. Yes ☐ 2. No

If yes, antibiotics given

☐ 1. 24 hours before ☐ 2. 3 days before ☐ 3. 7 days before

Intravenous infusion

☐ 1. Yes
☐ 2. No
☐ 3. Unknown

If yes, then how much?

☐ 1. Less than 500 ml per day
☐ 2. 500-1000 ml per day
☐ 3. >1000 ml per day

Time of intravenous infusion?

☐ 1. 24 hours
☐ 2. 3 days before
☐ 3. 7 days before

Pain medication

☐ 1. Yes
☐ 2. No

If yes for pain medication

☐ 1. PN pain medication
☐ 2. Regular pain medication
☐ 3. Both PN and regular pain medication

Non-opioid medication

☐ 1. Yes ☐ 2. No

Opioid medication

☐ 1. Yes ☐ 2. No

Benzodiazepine medication

☐ 1. Yes ☐ 2. No

If yes for benzodiazepine medication

☐ 1. PN medication
☐ 2. Regular medication
☐ 3. Both PN and regular medication

Tube feeding

☐ 1. Yes
☐ 2. No

If yes for tube feeding

☐ 1. Less than 500 ml per day
☐ 2. 500 - 1000 ml per day
☐ 3. >1000 ml per day

Oxygen therapy

☐ 1. Yes ☐ 2. No

Respiratory support

☐ 1. Yes ☐ 2. No

Nebulizer

☐ 1. Yes ☐ 2. No

Suctioning ☐ 1. Yes - suction of patient ☐ 2. No - decision for no suction ☐ 3. Not registered

Positioning of patient ☐ 1. Yes - turning and positioning the patient ☐ 2. Positioning not documented/unknown

Other treatments/medications ☐ 1. Scopoderm ☐ 2. Other (specify)

Other treatments – specify

6. Communication with relatives

Names of relatives documented ☐ 1. Yes ☐ 2. No ☐ 3. Unknown

Information about relatives documented ☐ 1. Family tree ☐ 2. Contact information of relatives ☐ 3. Unknown

When were relatives informed of the deteriorating status of the patient?

Relatives present close to death /at deathbed ☐ 1. Yes ☐ 2. No ☐ 3. Unknown

Health professionals meeting with relatives prior to death *(family meeting, formal or informal) in the 7 days before death.* ☐ 1. Yes ☐ 2. No ☐ 3. Unknown

Evidence of conversations with relatives about "*Care pathway for the dying*"

7. Health Care professionals involved in diagnosing dying

Health care professionals assessed patient's need for end-of-life care

☐ 1. Yes
☐ 2. No
☐ 3. Unknown

Who made the assessment?

☐ 1. Nurse
☐ 2. Physician
☐ 3. Another health professional

Where was the assessment documented?

☐ 1. Nurses' documentation
☐ 2. Physicians' documentation
☐ 3. "*Care pathway for the dying*"
☐ 4. Other

**Nurses documenting need for decision-making on end-of-life care**	
